# The complete nucleotide sequence of chloroplast genome of *Euphorbia kansui* (Euphorbiaceae), an endemic herb in China

**DOI:** 10.1080/23802359.2018.1495122

**Published:** 2018-08-01

**Authors:** Mi-Li Liu, Wei-Bing Fan, Ying Wu, Ying-Juan Wang, Zhong-Hu Li

**Affiliations:** Key Laboratory of Resource Biology and Biotechnology in Western China, Ministry of Education, College of Life Sciences, Northwest University, Xi’an, China

**Keywords:** Chloroplast genome, conservation, *Euphorbia kansui*, phylogenetic analysis

## Abstract

*Euphorbia kansui* T.N. Liou ex S.B. Ho (Euphorbiaceae) is a perennial herb plant endemic to China. This species has important economic and medicinal values. In this study, we first characterized the complete nucleotide sequence of chloroplast (cp) genome of *E. kansui* using the Illumina Hiseq platform. The cp genome was 161,061 bp in length, comprising of a large single copy (LSC) region of 91,288 bp, a small single copy (SSC) region of 17,086 bp, and two inverted repeat regions of 26,343 bp each. The cp genome contains 130 genes, including 86 protein-coding genes, 8 ribosomal RNAs (rRNAs), and 36 transfer RNAs (tRNAs). The phylogenetic analysis indicated that *E. kansui* was placed as a sister to the congeneric *Euphorbia esula*.

*Euphorbia kansui* T.N. Liou ex S.B. Ho (Euphorbiaceae) is a perennial herb plant endemic to Northern China, and have long been used as traditional Chinese medicinal materials (Wang et al. 2003; Yan et al. 2014). In recent years, overexploitation by human beings, as well as the rapid climate change, results in the increasingly declining and fragmenting of its natural populations. It is thus urgent to take effective strategies to conserve and mange this endangered herb species. In plant, chloroplast (cp) DNA provided valuable phylogenetic information, owning to its conserved genome structures and comparatively high substitution rates (Wu and Ge 2012). Herein, we first characterize the complete chloroplast genome of *E. kansui* based on the Illumina next-generation sequencing technology.

The fresh leave tissues from a single individual of *E. kansui* were collected from Hechuan Town (Shaanxi, China; 110.3389°E, 35.1353°N), and genomic DNA was isolated using a modified CTAB method (Doyle and Doyle 1987). DNA sample and voucher specimen (No. EKLZH2013516) of *E. kansui* were deposited in the Northwest University Museum (NUM). Then, the high quality DNAs were subjected to Illumina sample preparation, and pair-read sequenced were conducted on the Illumina Hiseq 2500 platform (Novogene Bioinformatics Technology Co., Ltd). In total, about 1,260,648 high-quality reads were obtained and used for the complete cp genome reference-guided assembly using the MIRA 4.0.2 (Chevreux et al. 2004) and MITObim version 1.7 (Hahn et al. 2013). The annotation of cp genome was conducted using the online program Dual Organellar Genome Annotator (DOGMA, Wyman et al. 2004), and then manually adjusted the positions of start codes, stop codes, introns, and exons by comparison with homologous genes in other chloroplast genomes. In addition, all tRNA genes were further verified online using the software tRNAscan-SE1.21 (Schattner et al. 2005). Eventually, the annotated cp genome sequence of *E. kansui* has been submitted to GenBank with the accession number MH392274, and circular genome maps were drawn using the program OGDRAW (Lohse et al. 2013).

The complete cp genome of *E. kansui* was a typical quadripartite circular molecule with a length of 161, 061 bp, which comprising a large single copy (LSC) region of 91, 288 bp and a small single copy (SSC) region of 17, 086 bp, separated by two inverted repeat regions (IRs) of 26,343 bp. The plastid genome contains 130 genes, including 86 protein-coding genes, 36 transfer RNAs (tRNAs), and 8 ribosomal RNAs (rRNAs). Among them, 19 genes duplicated in the IRs. A total of 13 genes (*atpF*, *rpoC1*, *rpl2*, *ndhB*, *ndhA*, *tRNA-Lys*, *tRNA-Leu*, *tRNA-Val*, *tRNA-Ala,* and *tRNA-Ile*) contained one intron, and two genes (*ycf3*, *rps12*) contained two introns. The overall GC content of *E. kansui* plastome is 35.5%, while the corresponding values of LSC, SSC, and IR regions are 32.6%, 30.2%, and 42.4%, respectively.

To investigate the phylogenetic position of *E. kansui*, the cp genomes of 19 species within order Malpighiales were downloaded from NCBI. All of the 20 complete plastome sequences were aligned using the software MAFFT (Katoh and Standley 2013) with the default parameters. The phylogenetic analysis was conducted using the program MEGA6 (Tamura et al. 2013) with 1000 bootstrap replicates. The results indicated that *E. kansui* was placed as a sister to the congeneric *Euphorbia esula* with high bootstrap value (Figure 1).

**Figure 1.**
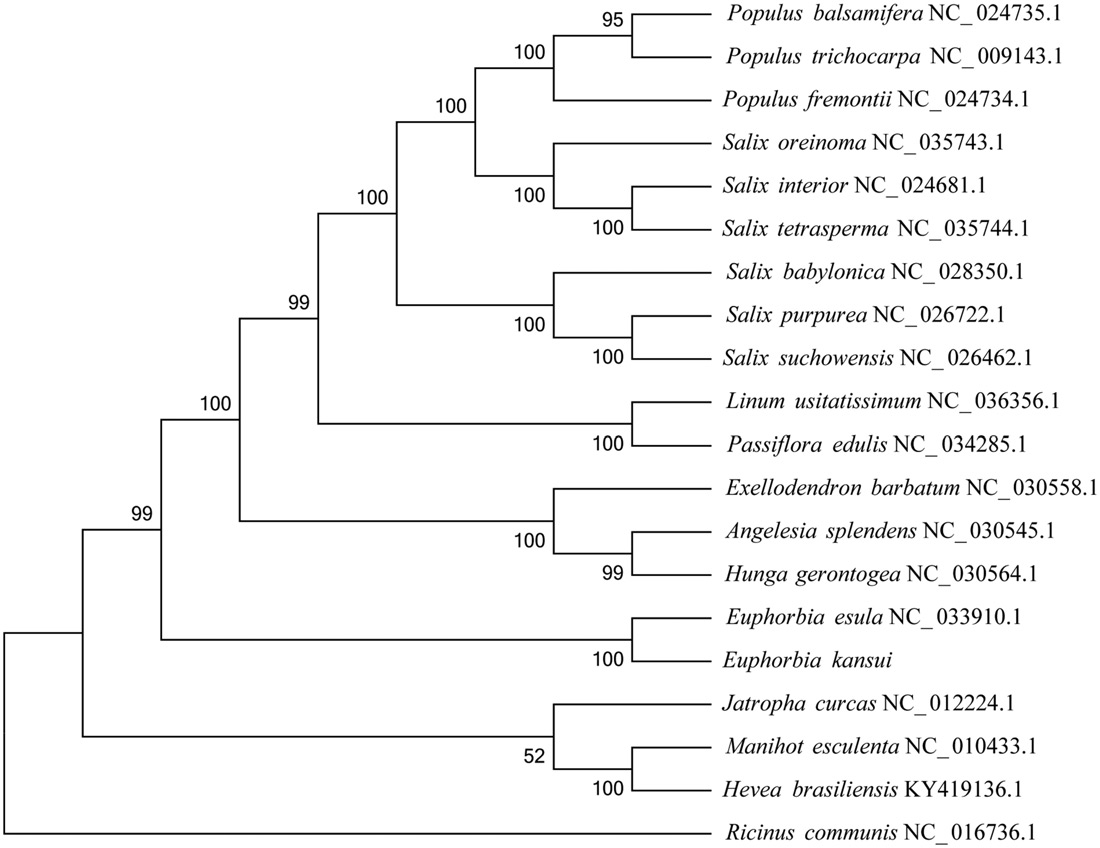
Phylogenetic tree based on twenty complete chloroplast genome sequences.
